# The Ibr-7 derivative of ibrutinib radiosensitizes pancreatic cancer cells by downregulating p-EGFR

**DOI:** 10.1186/s12935-020-01548-6

**Published:** 2020-09-17

**Authors:** Biqin Tan, Rong Dong, Bo Zhang, Youyou Yan, Qingyu Li, Fei Wang, Nengming Lin

**Affiliations:** 1 Department of Clinical Pharmacology, Key Laboratory of Clinical Cancer Pharmacology and Toxicology Research of Zhejiang Province, Affiliated Hangzhou First People’s Hospital, Zhejiang University School of Medicine, Hangzhou, 310006 Zhejiang China; 2 Translational Medicine Research Center, Affiliated Hangzhou First People’s Hospital, Zhejiang University School of Medicine, Hangzhou, 310006 Zhejiang China

**Keywords:** Pancreatic cancer, Ibr-7, Radiosensitivity, Apoptosis, EGFR

## Abstract

**Background:**

Radiotherapy is one of the main treatments for pancreatic cancer, but radiation resistance limits its clinical application. As a result, novel therapeutic agents to improve radiosensitivity are urgently needed. This study aimed to investigate the effect of Ibr-7 (a derivative of ibrutinib) on the radiosensitivity of human pancreatic cancer cells.

**Methods:**

The effect of Ibr-7 on pancreatic cancer cell proliferation was detected by CCK-8 assays. Radiosensitivity was assessed by clonogenic formation assays. Cell cycle and cell apoptosis were analysed by flow cytometry. DNA damage was evaluated by immunofluorescence analysis. The expression levels of PARP, Cleaved caspase 3, p-EGFR and EGFR were determined by western blot.

**Results:**

Ibr-7 showed an anti-proliferative effect on PANC-1 and Capan2 cells in a dose- and time-dependent manner. Ibr-7 (2 μmol/L) enhanced the effect of radiation on PANC-1 and Capan2 cells. Further findings showed that this combination enhanced G2/M phase arrest and increased cell apoptosis. Additional molecular mechanism studies revealed that the expression of p-EGFR was decreased by Ibr-7 alone or in combination with radiation. Overexpression of p-EGFR reversed the cell apoptosis induced by Ibr-7 combined with radiation. Moreover, the expression of γ-H2AX was significantly decreased in the Ibr-7 plus radiation group.

**Conclusions:**

Our study indicated the potential application of Ibr-7 as a highly effective radiosensitizer for the treatment of pancreatic cancer cells.

## Background

Pancreatic cancer is a highly invasive and fatal disease, with a low 5-year survival rate of only 5% [1]. Pancreatic cancer is diagnosed in the middle or terminal stage, and 80% of patients are ineligible for surgery. The treatment options for these patients are chemotherapy and radiation therapy [2, 3]. Gemcitabine is the first-line treatment drug for pancreatic cancer. FOLFIRINOX (fluorouracil, leucovorin, irinotecan, and oxaliplatin) regimens are available for improving progression-free survival (PFS) and overall survival (OS). Approximately 70% of pancreatic cancer patients receive radiotherapy, either alone or in combination with chemotherapy [4, 5]. However, many patients do not benefit from such cytotoxic drugs alone or together with radiotherapy. Pancreatic cancer cells inevitably develop radioresistance, which is one of the main causes of the poor prognosis of patients in clinical treatment [6–8]. Exploring new targeted agents to improve the radiosensitivity of pancreatic cancer is critical.

Ibrutinib is a small molecule inhibitor that targets Bruton’s tyrosine kinase [9]. Ibrutinib binds to Cys481 (a cysteine residue), forms a complex with BTK, and inhibits the enzymatic activity of BTK. Targeting BTK restores T cell-dependent antitumour immune responses, inhibiting tumour growth. Ibrutinib exhibited potent antitumour effects against various cancers, including lymphoma and solid tumours (such as non-small cell lung cancer and pancreatic cancer) [10–14]. However, our previous research showed that the antitumour effect of ibrutinib was limited to blood cancer cells [15]. The clinical trials of ibrutinib in solid tumours are facing challenges. Based on these findings, we synthesized a series of ibrutinib derivatives, of which Ibr-7 exhibited robust antitumour activity towards NSCLC and pancreatic cancer [15, 16]. In addition, our previous research showed that ibrutinib (10 μmol/L) enhanced the effects of radiation therapy [17]. Therefore, exploring the radiosensitizing effect of Ibr-7 on pancreatic cancer cells is of great interest.

In the present study, evaluated the radiosensitizing effect of Ibr-7 in pancreatic cancer cell lines. Our results suggested that Ibr-7 sensitized the pancreatic cancer cell lines PANC-1 and Capan2 to radiation therapy. Further study indicated that Ibr-7 induced G2/M cell cycle arrest and cell apoptosis. Ibr-7 efficiently inhibited p-EGFR expression, similar to that of ibrutinib. However, unlike ibrutinib, Ibr-7 did not suppress the phosphorylation of the AKT/mTOR signalling pathway. These results may offer an effective strategy for enhancing the effect of radiation therapy and will provide insights into the development of ibrutinib derivatives.

## Materials and methods

### Cell lines and reagents

The human pancreatic cancer cell lines PANC-1 and Capan2 were purchased from the Stem Cell Bank of Type Culture Collection of the Chinese Academy of Sciences (Shanghai, China). PANC-1 and Capan2 cells were cultured in DMEM or RPMI-1640 medium supplemented with 10% FBS in an atmosphere of 5% CO2 at 37 °C. Ibrutinib (PCI-32765, 99.2%) was purchased from Selleck, USA. Ibr-7 (Lot: 20161216, > 95%) was provided by Hangzhou Hezheng Pharmaceutical Co., Ltd. Ibrutinib and Ibr-7 were dissolved in DMSO (Sigma-Aldrich, St Louis, Missouri, USA) at a concentration of 100 mmol/L and stored at − 20 °C.

### Antibodies and reagents

Anti-PARP (46D11, 9532, 1:1000), anti-cleaved caspase 3 (CST, D175R, 9661S, 1:1000), anti-caspase 3 (CST, 9662S, 1:1000), anti-EGFR (4267 s, 1:1000), and anti-p-EGFR (Tyr1068; 3777S, 1:1000) were purchased from Cell Signaling Technology (Danvers, MA, USA). β-actin (J0914, 1:2000) was purchased from Santa Cruz Biotechnology (Dallas, TX, USA). PVDF (0.45 μm, Millipore, Cat. No.: IPVH00010), 5× loading buffer (Biosharp, BL502b), peroxidase-conjugated goat anti-mouse IgG (H + L; BL001A) and peroxidase-conjugated goat anti-rabbit IgG (H + L; BL003A) were purchased from Biosharp. ECL HRP substrates for western blot were purchased from Cyanagen (WESTAR ηC2.0, lot HG13A-CC).

### Cell viability assay

Cell proliferation was measured by the Cell Counting Kit-8 (CCK-8, Bestbio, Shanghai, China) assay. Ibrutinib or Ibr-7 was diluted 1:1000 with serum-free medium to a final concentration of 100 μmol/L for the CCK-8 assay. Cells were cultured in 96-well plates at 3–5 × 10^3^/well. Cells were treated with Ibr-7 (1.56–25 μmol/L) or ibrutinib (1.56–50 μmol/L) by the half dilution method for 24, 48 or 72 h. The cells were measured using CCK-8 solutions at 450 nm by a SpectraMax M2e (Molecular Devices, San Jose, CA, USA). Cell viability was calculated for each well, and the 50% growth inhibition concentration (IC50) was determined. Assays were performed on at least three independent experiments.

### Clonogenic assay

The pancreatic cancer cells were plated in six-well plates and pretreated with Ibr-7 or DMSO as a control for 24 h. Then, the cells were exposed to the indicated doses (0, 2, 4, and 6 Gy) of radiation. The cells were then incubated with DMEM or RPMI-1640 medium supplemented with 10% FBS at 37 °C in a 5% CO2 atmosphere. After 14 days, the colonies were stained with 0.1% crystal violet (Sigma-Aldrich) in absolute methanol for 30 min. Colonies containing more than 50 cells were counted. The survival fraction (SF) was calculated as previously described [18]. SF = mean number of colonies/(plating efficiency × number of cells inoculated) in the treated groups, and the plating efficiency = (mean number of colonies/number of cells inoculated) in the untreated groups. A multitarget click mathematical model was applied to determine the cell survival fraction. We then calculated the sensitization enhancement ratio (SER) as the dose (2 Gy) of radiation divided by the dose (2 Gy) of radiation plus Ibr-7 (SER = SFIR/SFIR + Ibr-7). The mean lethal dose of cells (D0), extrapolation number (N) values, and quasithreshold dose (Dq) were also calculated according to the curve. Error bars were calculated as SDs of the results of three independent experiments.

### Cell cycle analysis

PANC-1 and Capan2 cells were divided into four groups: the control group (DMSO), the Ibr-7 (2 μM) group, the radiation (2 Gy) group, and the combination group. A total of 5 × 10^5^ cells were plated into 6-well plates, exposed to Ibr-7 for 24 h, and then exposed to 6-MV of X-ray. After another 24 h, the cells were trypsinized and fixed with precooled 70% ethanol at − 20 °C overnight. Then, PI (supplemented with RNase A) was added to the cells and incubated for 30 min at room temperature. The treated cells were analysed by flow cytometry (Becton–Dickinson, Franklin Lakes, NJ, USA). Error bars were calculated as SDs of the results of three independent experiments.

### Apoptosis assay

Cells were divided into four groups as described above. The cells were plated into 6-well plates, pretreated with Ibr-7 for 24 h and exposed to 2 Gy of radiation. After another 24 h, the cells were trypsinized and washed twice with PBS. Then, the cell suspension was stained with 5 μL of Annexin V-FITC and 5 μL of PI solutions and analysed by flow cytometry (Becton–Dickinson, Franklin Lakes, NJ, USA). Error bars were calculated as SEs of the results of three independent experiments.

### Immunofluorescence analysis

A total of 3 × 10^4^ cells were plated on coverslips in 24-well plates and pretreated with DMSO or Ibr-7 for 24 h. Then, the cells were exposed to radiation (6 Gy). The cells were then incubated for 24 h and collected. The cells were washed in PBS 3 times and fixed with 4% paraformaldehyde for 30 min. After cells were permeabilized with 0.5% Triton-100 for 15 min, the cells were blocked with 1% BSA and then incubated with anti-γ-H2AX antibody overnight at 4 °C. The cells were incubated with goat anti-rabbit IgG (FITC-labelled) antibodies (Beyotime) for 60 min, followed by incubation with DAPI for 5 min. Finally, the cells were photographed with an immunofluorescence microscope (Olympus IX53).

### Western blot analysis

Cultured cells were collected and washed with PBS three times. Total protein extraction was conducted using RIPA lysis buffer containing phosphatase and protease inhibitors according to the manufacturer’s instructions. A total of 20–40 μg of protein was subjected to 10% SDS/PAGE and transferred to PVDF membranes. The membranes were then blocked with 5% skim milk for 1 h at room temperature. The membranes were incubated with primary antibodies against PARP, cleaved caspase 3, EGFR, p-EGFR and β-actin overnight at 4 °C and then incubated with secondary antibodies for 1 h at room temperature. The membranes were washed with TBST 3 times, and the proteins were visualized by adding ECL reagent.

### Plasmid transfection

The pcDNA3.1-EGFR plasmid, phosphomimic mutant of EGFR plasmid and empty vector were purchased from GenePharma. The plasmid was transfected into PANC-1 and Capan2 cells with jetPRIME reagent according to the manufacturer’s protocol as below. Cells are seeded in 6-well plates 24 h prior to transfection. Dilute 2 μg DNA into 200 μL jetPRIME buffer. Mix by vortexing. Add 4 μL jetPRIME, vortex for 10 s, spin down briefly. Incubate for 10 min at RT. Add 200 μL of transfection mix per well drop wise onto the cells in serum containing medium, and distribute evenly. Gently rock the plates back and forth and from side to side. Replace transfection medium after 4 h by cell growth medium and return the plates to the incubator. Analyze after set time.

### Statistical analysis

The results were generated from at least three independent experiments. All quantitative values are presented as the mean ± SD (standard deviation). Student’s *t* test was used to determine the significance, and statistical significance was defined as *p *< 0.05. Graphs were generated using GraphPad software.

## Results

### Ibr-7 inhibited pancreatic cancer cell growth

**Fig. 1:**
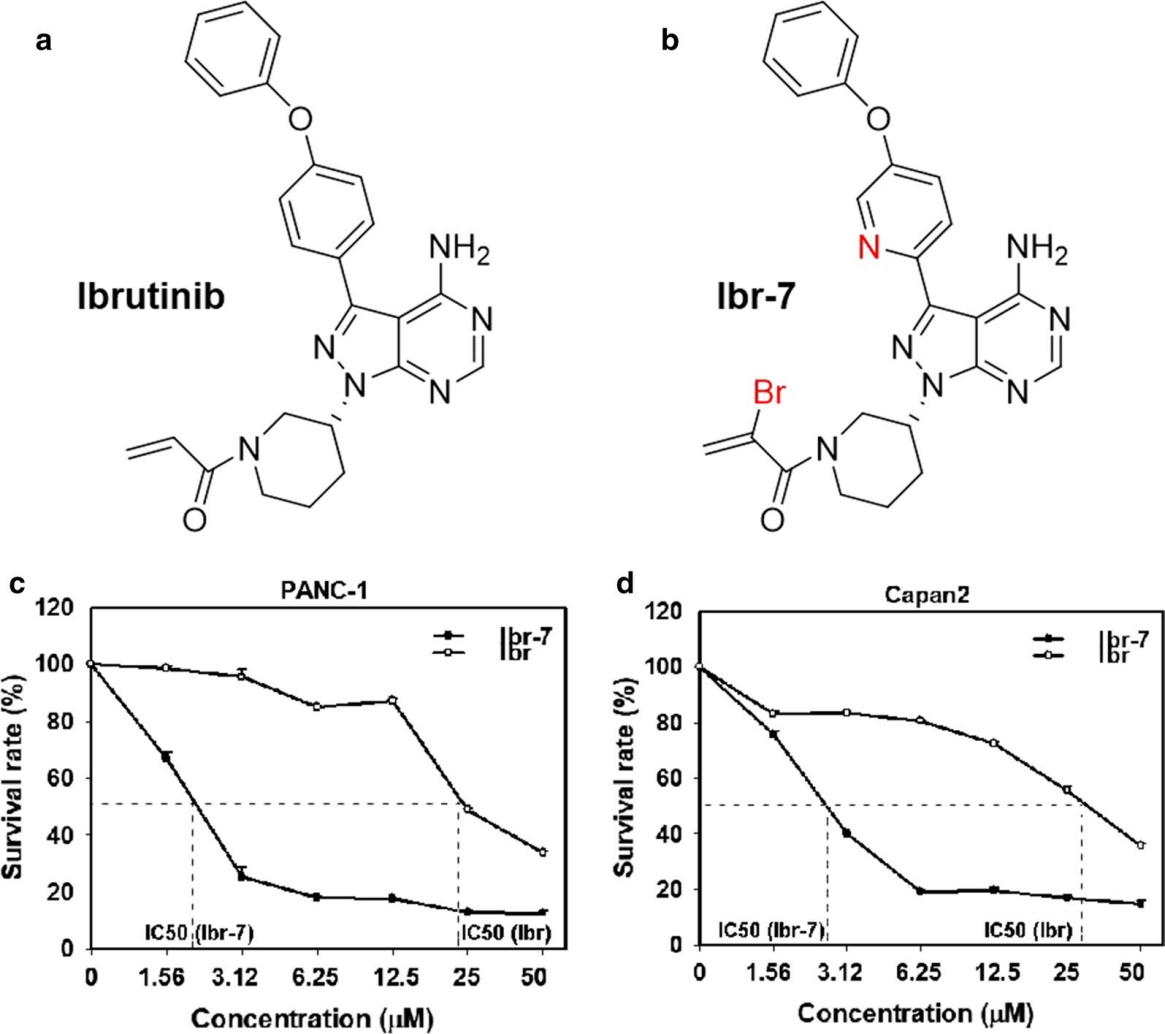
Ibr-7 showed potent anti-proliferative activity against pancreatic cancer cells in dose-dependent manner. **a** The chemical structure of ibrutinib (Ibr) and Ibr-7. **b** CCK8 assay was performed to detect the inhibitory activity of ibrutinib (Ibr) or Ibr-7 on the proliferation on pancreatic cancer cell line PANC-1, Capan2 cell lines for 48 h in vitro. Three independent experiments were performed and data were presented as mean ± SD (standard deviation)

Ibr-7 is a synthesized derivative of ibrutinib that exhibited antitumour effects against various cancer cells compared with those of the parental compound (Fig. 1a, b) [15]. To determine the anti-proliferative effect of Ibr-7 in pancreatic cancer cells, both PANC-1 and Capan2 cell lines were treated with increasing doses of ibrutinib or Ibr-7 (1.56–50 μmol/L) for 24 h, 48 h or 72 h, and changes in cell proliferation were examined by CCK-8 assays. Our data demonstrated that cell viability was markedly reduced by Ibr-7 in both dose- and time-dependent manners (Additional file 1: Fig. S1, Additional file 2: Table S1). The IC50 values of Ibr-7 at 48 h of treatment were 1.7 μmol/L and 2.5 μmol/L, while the IC50 values of ibrutinib were 20.8 μmol/L and 29.6 μmol/L for PANC-1 and Capan2, respectively (Fig. 1, Table 1). For both cell lines, the IC50 values of Ibr-7 were approximately one-tenth of the IC50 values of ibrutinib, indicating that Ibr-7 exhibits more effective antitumour activity than ibrutinib.

**Table 1:** The IC50 of Ibr-7 and ibrutinib in PANC-1 and Capan2 cells

Cell line	Ibrutinib (μmol/L)	Ibr-7 (μmol/L)
PANC-1	20.8	1.7
Capan2	29.6	2.5

### Ibr-7 pretreatment enhanced the radiosensitivity of pancreatic cancer cells

**Fig. 2:**
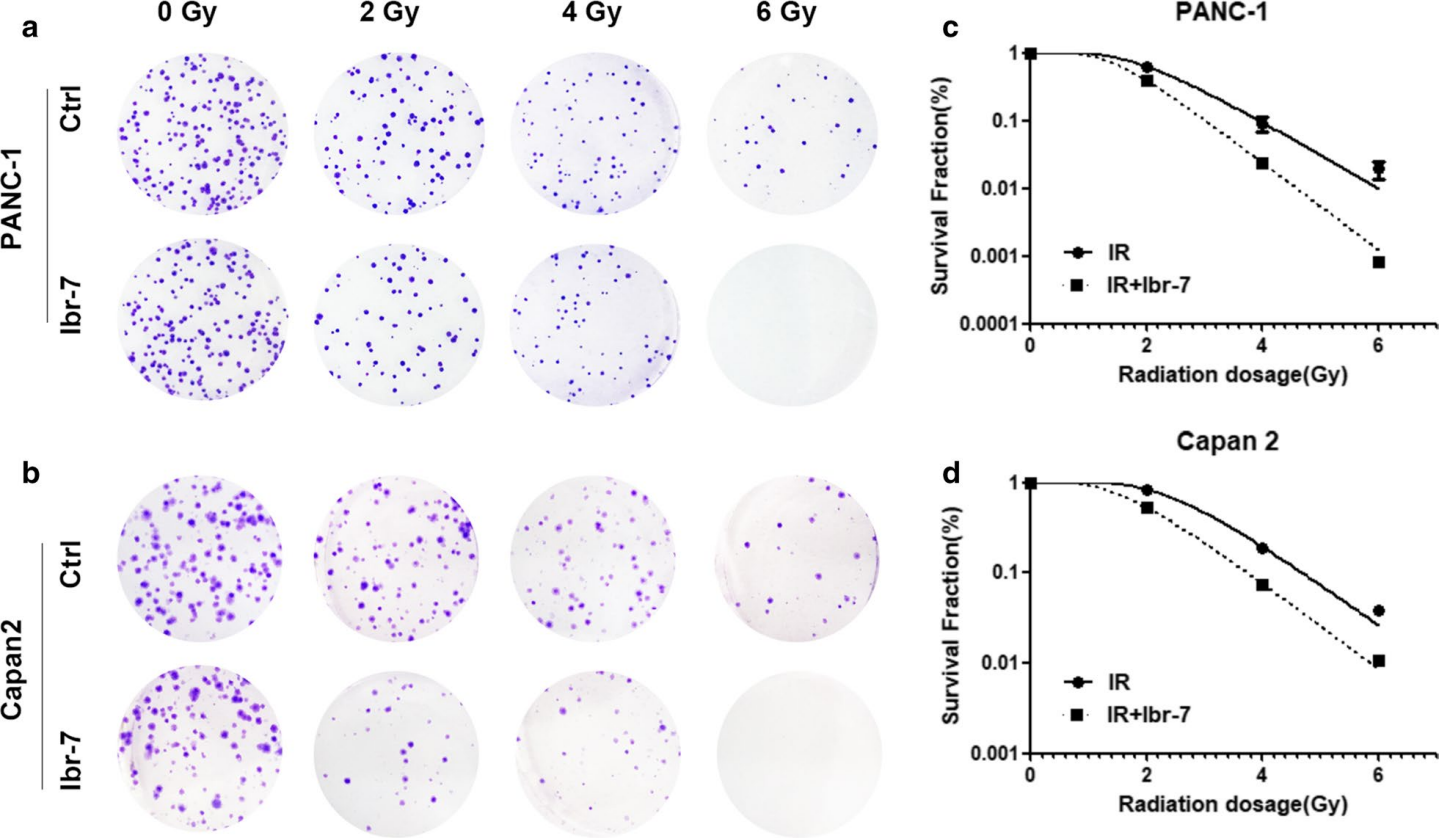
Ibr-7 sensitized pancreatic cancer cells to radiation. A representative image of colony formation in PANC-1 and Capan2 cells treated with radiation alone or with Ibr-7 (2 μmol/L) and radiation (0, 2, 4, 6 Gy) (**a**, **b**). Clonogenic cell survival assays were performed for PANC-1 and Capan2 cells that were treated with Ibr-7 (2 μmol/L) or DMSO for 24 h, then were exposed to different doses of γ-radiation. A multi-target click mathematical model simulated the cell survival fraction curve (**c**, **d**). Results shown are the mean ± SD of 3 independent experiments. Significance was determined by Student’s *t*-test (*p < 0.05 compared with combination group)

Based on the effect of Ibr-7 on pancreatic cancer cells, we further investigated the radiosensitizing effect of Ibr-7 with clonogenic survival assays. Cells were seeded into 6-well plates, treated with 2 μmol/L Ibr-7 and then exposed to IR (2, 4, or 6 Gy). The survival curve was derived from the single-hit multitarget model [${\text{Y}} = 1 - \left( {1 - {\text{e}}^{{ - {\text{k*x}}}} } \right)^{\text{N}}$]. The sensitization enhancement ratio (SER) was measured according to the model. As shown in Fig. 2 and Table 2, pretreatment with Ibr-7 enhanced the radiosensitivity of both PANC-1 (SER = 1.63) and Capan2 cells (SER = 1.59).

**Table 2:** The radiosensitization activity of PANC-1 and Capan2 cells treated with or without Ibr-7

	K	N	D0/Gy	Dq/Gy	SF2	SERSF2
PANC-1						
IR	1.134	9.097	0.88	1.94	63.05 ± 0.08	–
IR + Ibr	1.492	9.432	0.67	1.50	38.69 ± 0.02*	1.63
Capan2						
IR	1.044	13.89	0.96	2.53	84.20 ± 0.02	–
IR + Ibr	1.105	6.462	0.90	1.68	52.85 ± 0.01***	1.59

K, a passivation constant, derived directly from the fitting equation; N, extrapolation number, derived directly from the fitting equation; D0, mean lethal dose; Dq, quasithreshold dose; SF2, survival fraction (2 Gy); SER, sensitization enhancement ratio

* p < 0.05, *** p < 0.001

### Ibr-7 promoted G2/M cell cycle arrest of pancreatic cancer cells after radiation

**Fig. 3:**
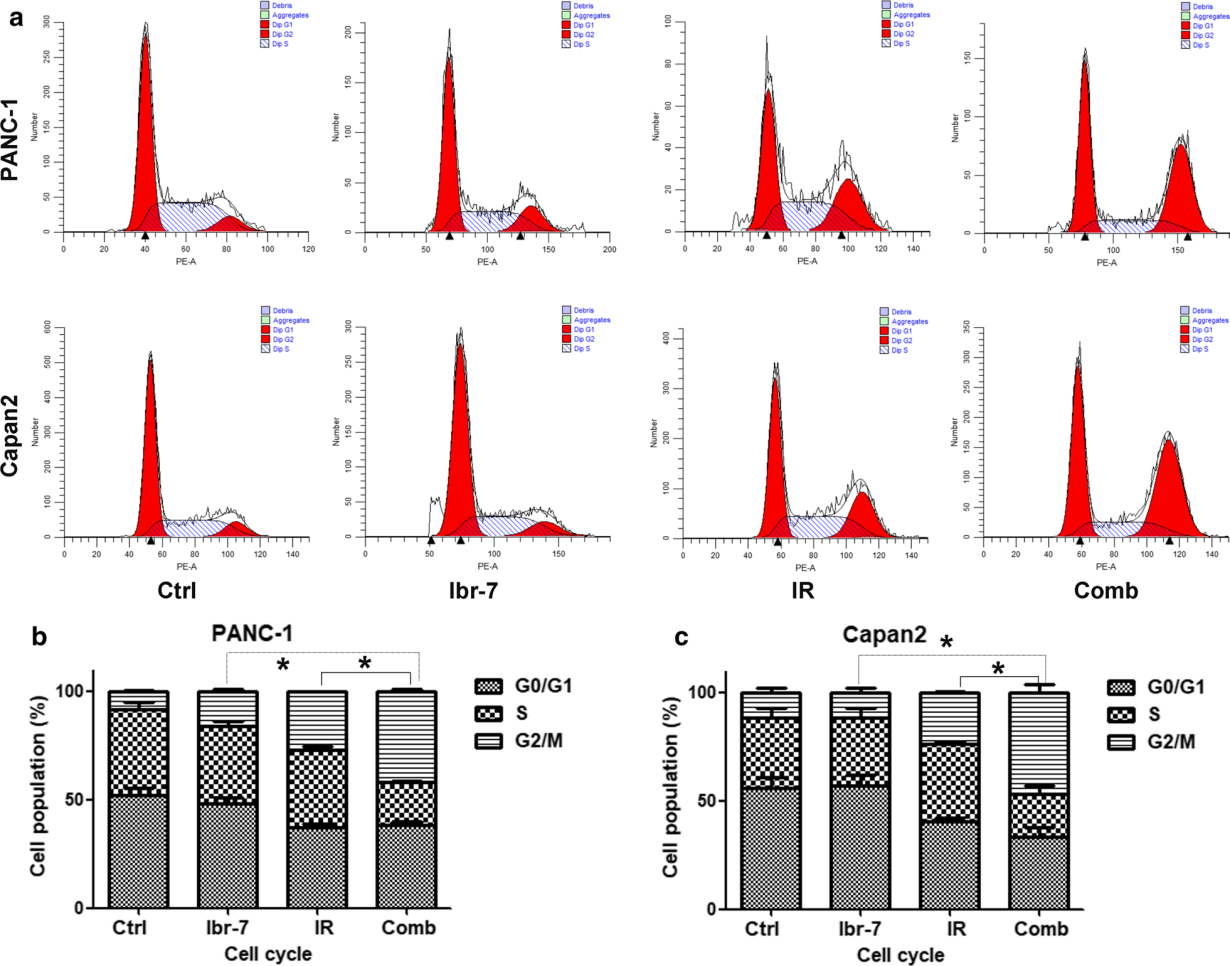
Ibr-7 promoted radiation-induced G2/M phase arrest in pancreatic cancer cells. **a** Representative histograms showing the cell cycles of PANC-1 and Capan2 cells in the Ctrl, Ibr-7, radiation and combination groups. Cells were treated with Ibr-7 (2 μmol/L) for 24 h, exposed to 6 Gy of radiation and stained with PI after another 24 h. **b** The percentages of the cell cycle phases in each group were quantified. The results shown are the mean ± SD of 3 independent experiments. Significance was determined by Student’s *t*-test (*p < 0.05 compared with the combination group)

To identify whether Ibr-7 could alter radiation-induced cell cycle distribution, PI staining assays of PANC-1 and Capan2 cells were performed. PANC-1 and Capan2 cells were treated with 2 μmol/L Ibr-7 for 24 h and then irradiated with 6 Gy. The cells were collected after 24 h. As shown in Fig. 3, pretreatment with Ibr-7 showed little effect on the G2/M phase (16.12% ± 0.88% *vs* 8.32% ± 0.48%) in PANC-1 cells compared with that of the controls. The same results were observed in Capan2 cells (11.43% ± 2.07% *vs* 11.54% ± 1.87%). Cells exposed to radiation alone exhibited G2/M phase arrest compared with that of control cells. Compared with that of the IR group, a significant increase in G2/M accumulation was observed in both PANC-1 (41.93% ± 1.07% vs 27.17% ± 0.04%, *p < 0.05) and Capan2 cells (46.6% ± 3.5% vs 23.92 ± 0.22%, *p < 0.05) in response to the combination treatment. Therefore, Ibr-7 increased radiation-induced G2/M arrest in pancreatic cancer cells.

### Ibr-7 increased IR-induced apoptosis in pancreatic cancer cells

**Fig. 4:**
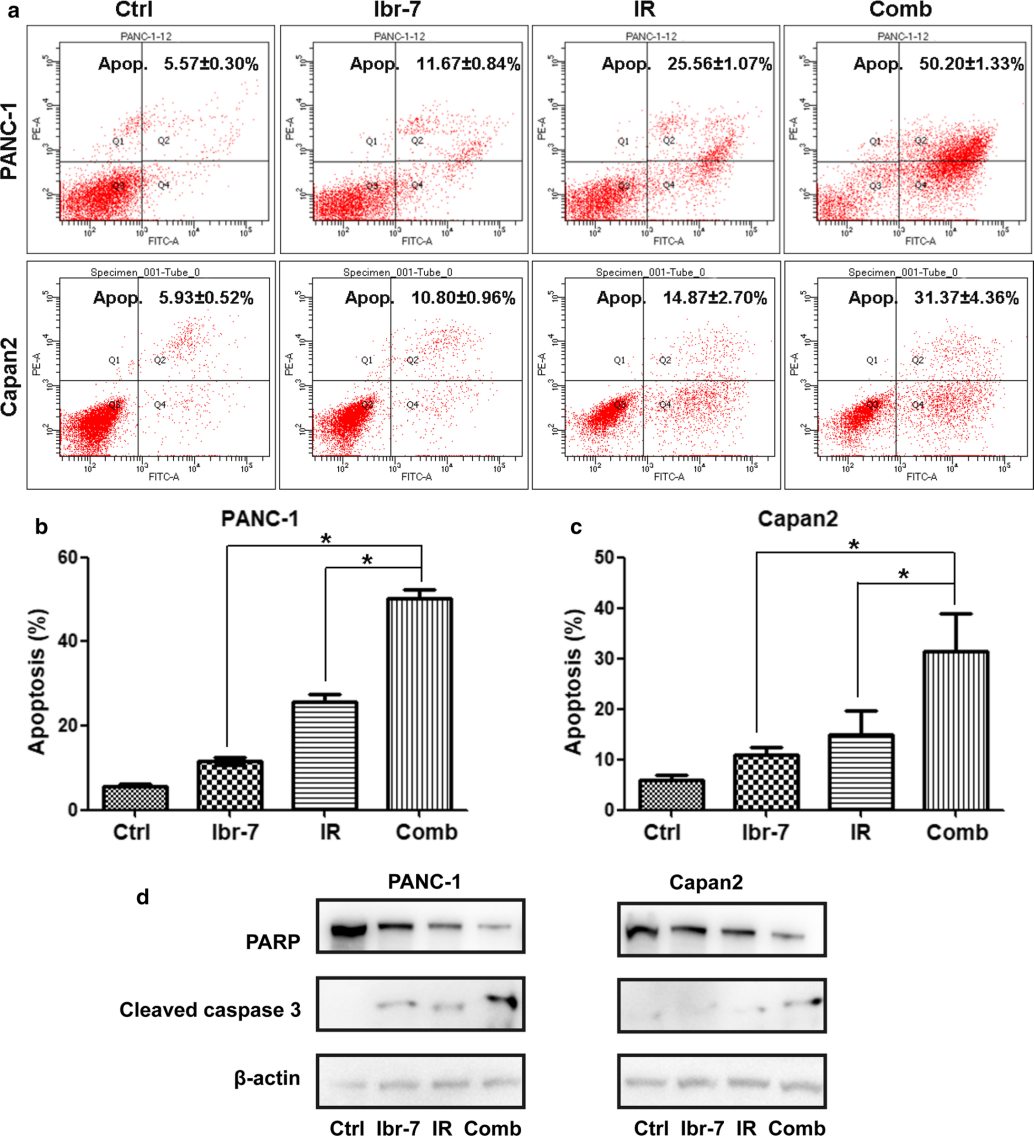
Ibr-7 induced cell apoptosis in irradiated PANC-1 and Capan2 cells. **a** Representative histograms showing the apoptosis of PANC-1 and Capan2 cells in the Ctrl, Ibr-7, radiation and combination groups. Cells were treated with Ibr-7 (2 μmol/L) for 24 h, exposed to 6 Gy of radiation and stained with Annexin V-FITC and PI after another 24 h. **b** The percentages of apoptotic cells in each group were quantified. **c** Cells were pretreated with Ibr-7 for 24 h and then exposed to radiation for another 24 h. Western blot analysis was performed to detect the expression of PARP, Cleaved caspase 3, β-actin was used as a loading control. **d** The ratio of PARP/β-actin, Cleaved caspase 3/β-actin was quantified by densitometry based on immunoblot images. The results shown are the mean ± SE of 3 independent experiments. Significance was determined by Student’s *t*-test (*p < 0.05 compared with the combination group)

To further investigate the potential mechanisms by which Ibr-7 enhances radiosensitivity, we next conducted an apoptosis assay by Annexin V-conjugated FITC and propidium iodide (PI) staining. The total apoptosis rate was calculated. The percentage of apoptotic cells in Ibr-7 combined with radiation group (50.2 ± 1.33%) exhibited a markedly increased compared to Ibr-7 (11.67 ± 0.84%) or radiation (25.56 ± 1.07%) alone (Fig. 4a, b, *p < 0.05). In Capan 2 cells, the results were the same (Fig. 4a, c, *p < 0.05). To validate these results, western blot was conducted to measure the expression of apoptosis-related proteins. The expression of PARP was decreased after exposure to Ibr-7 and radiation, while cleaved caspase 3 was increased (Fig. 4d). In summary, these results indicate that Ibr-7 enhances radiation-induced apoptosis.

### Ibr-7 combined with radiation increased DNA damage in pancreatic cancer cells

**Fig. 5:**
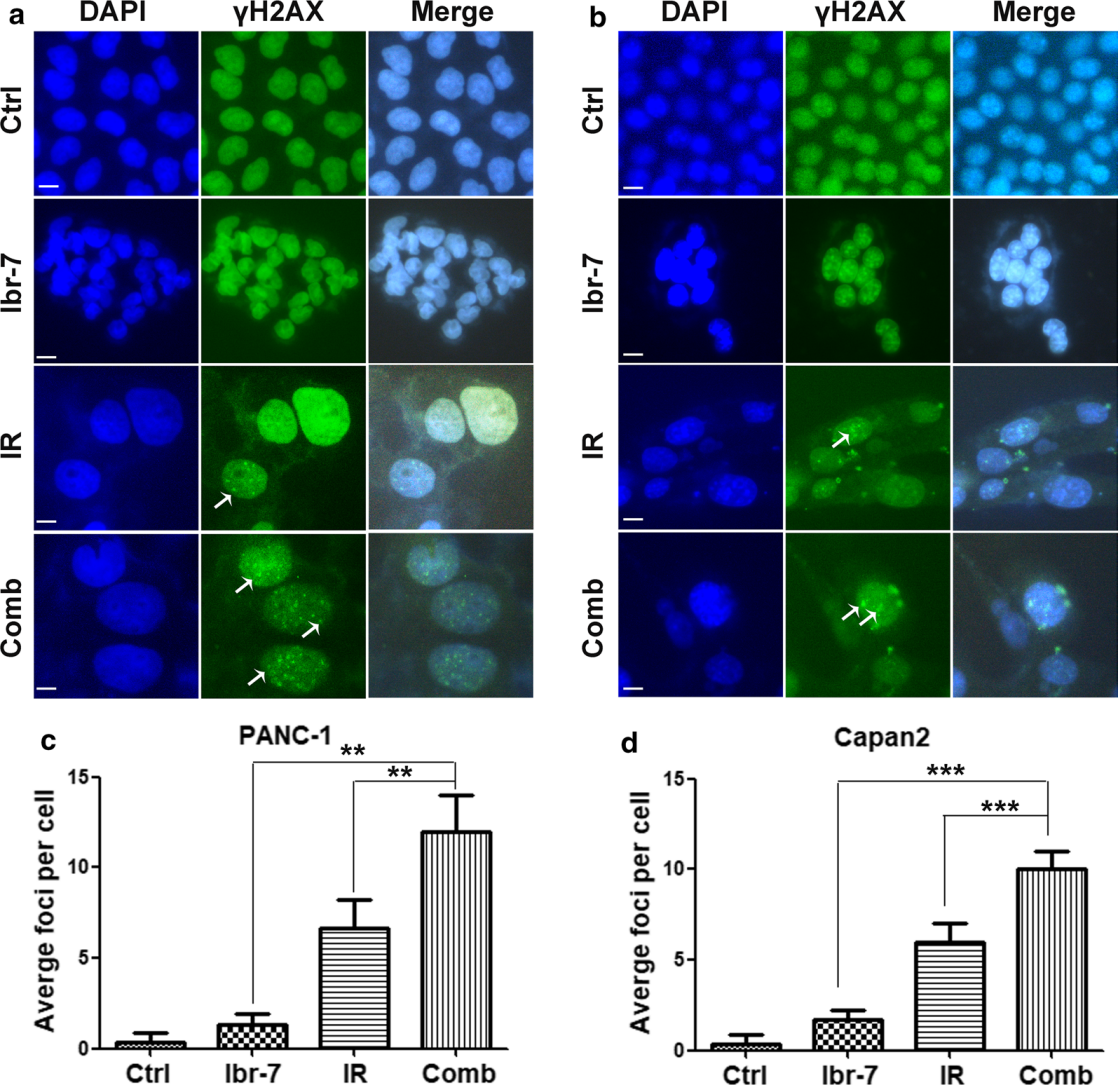
Ibr-7 increased radiation induced DNA double-strand breaks. **a** The representative images of γ-H2AX foci immunofluorescence staining in PANC-1 and Capan2 cells treated with 6 Gy radiation with or without Ibr-7 at 24 h after radiation. **b** Cells with γ-H2AX foci were counted and quantified. Error bars indicated SE. Significance was determined by Student’s *t*-test. Scale bar = 10 μm. (**p < 0.01, ***p < 0.001 compared with combination group)

The major impact of radiation in cells is the induction of DNA double strand breaks (DSBs) and stimulation of DNA damage repair. Moreover, γ-H2AX is a rapid and sensitive cellular biomarker of DSBs [19]. γ-H2AX (phosphorylated at C-terminal serine residue 139) forms nuclear foci in the regions of nascent DSBs and subsequently undergoes dephosphorylation after the repair of DSBs. Therefore, the number of γ-H2AX foci is used as an indicator of the relative amount of DSBs and repair. To determine whether Ibr-7 could enhance radiation-induced DNA damage, we conducted immunofluorescence analysis to evaluate γ-H2AX foci. As shown in Fig. 5, Ibr-7 significantly increased the number of γ-H2AX foci per cell at 24 h following 6 Gy radiation compared to that of radiation alone (12.0 ± 2.0 compared to 6.67 ± 1.53 in PANC-1 cells and 10.0 ± 1.0 compared to 6.0 ± 1.0 in Capan2 cells, *p < 0.05). These results suggested that the combination of Ibr-7 and radiation increased DNA damage.

### Ibr-7 enhanced pancreatic cancer cell radiosensitivity in a p-EGFR-dependent manner

**Fig. 6:**
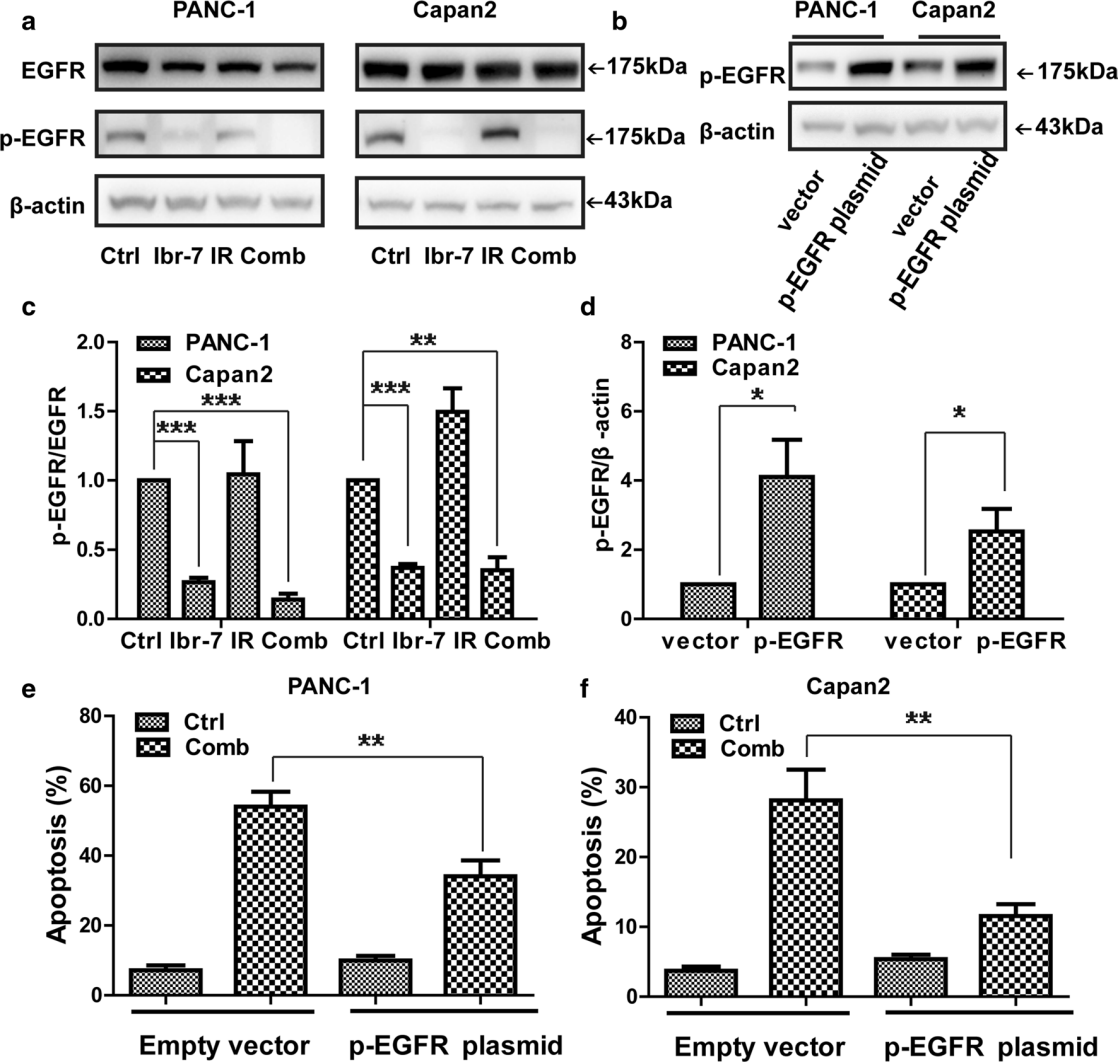
Involvement of p-EGFR in Ibr-7 sensitive radiation induced apoptosis in pancreatic cancer cells. **a** Cells were pretreated with Ibr-7 for 24 h and then exposed to radiation for another 24 h. Western blot analysis was performed to detect the expression of p-EGFR, EGFR, β-actin was used as a loading control. **b** Both PANC-1 and Capan2 cells were transfected with phosphomimic mutant of EGFR plasmid and empty vector. Cells were collected after 24 h transfection and the expression of p-EGFR were detected by western blot. **c** The ratio of p-EGFR/EGFR was quantified by densitometry based on immunoblot images. **d** The ratio of p-EGFR/β-actin was quantified by densitometry based on immunoblot images. **e**, **f** The ratio of apoptosis in PANC-1 and Capan2 cells that had been transfected with phosphomimic mutant of EGFR plasmid or empty vector, then pretreated with Ibr-7 and exposed to radiation. The percentages of cell apoptosis were quantified. Results shown are the mean ± SD of 3 independent experiments. Significance was determined by Student’s t-test (*p < 0.05, **p < 0.01, ***p < 0.001)

Downregulation of p-EGFR was considered to play a major role in the effect of ibrutinib. We therefore examined whether p-EGFR was the major target of the ibrutinib derivative Ibr-7 in PANC-1 and Capan2 cells after radiation treatment. As expected, the expression of p-EGFR was markedly decreased in response to Ibr-7 alone or in combination with radiation (Fig. 6a, c, **p < 0.01, ***p < 0.001), indicating the involvement of EGFR in the effect of Ibr-7 combined with radiation on PANC-1 and Capan2 cells.

Based on these results, we further analysed whether the downregulation of p-EGFR was required for apoptosis induced by Ibr-7 plus radiation. We transfected the phosphomimic mutant of EGFR plasmid and measured apoptosis. p-EGFR overexpression significantly decreased apoptosis in PANC-1 (34.10 ± 4.54 compared to 54.03 ± 4.26, **p < 0.01) and Capan2 cells (11.53 ± 1.70 compared to 28.1 ± 4.40, **p < 0.01) in response to the combination treatment (Fig. 6b, d–f). Meanwhile, we also transfected the EGFR plasmid. Similarly, EGFR overexpression significantly decreased apoptosis in PANC-1 (22.00 ± 5.09 compared to 41.30 ± 2.12) and Capan2 cells (12.60 ± 3.96 compared to 29.95 ± 2.47) in response to the combination treatment (Additional file 3: Fig. S2). Taken together, these results suggest that inhibiting p-EGFR might contribute to Ibr-7-induced radiosensitivity.

## Discussion

Our findings demonstrate that Ibr-7 strongly enhances the radiosensitivity of pancreatic cancer cells. These effects of Ibr-7 are attributable to activating G2/M arrest and inducing apoptosis, which resulted from the decreases expression of p-EGFR.

Ibrutinib has shown antitumour effects on chronic lymphocytic leukaemia [20–23]. Ibrutinib also inhibited growth in solid cancers, but the results were limited [24–27]. Based on these findings, a series of derivatives were developed. Ibr-7 is a promising candidate and exhibits robust antitumour activity against NSCLC and pancreatic cancer [15, 16]. Moreover, ibrutinib (10 μmol/L) was shown to induce radiosensitivity in pancreatic cancer cell lines in our previous study [17]. In the present study, the radiosensitizing effect of Ibr-7 on pancreatic cancer cells was evaluated. The IC50 values of Ibr-7 against PANC-1 and Capan2 cells were 1–2 μmol/L. At a concentration of 2 μmol/L, Ibr-7 robustly induced radiosensitivity in pancreatic cancer cells.

To explore the underlying molecular mechanisms responsible for the effect of Ibr-7, we first analysed the molecular structure of Ibr-7 and the relationship between ibrutinib and Ibr-7. Due to the similar structure of BTK, ibrutinib forms a complex with BTK and then inhibits BTK activity. The data also showed that ibrutinib inhibits the expression of EGFR, HER2/neu, and Her4/ErbB4. As a derivative of ibrutinib, Ibr-7 could also inhibit EGFR in NSCLC [28]. Whether Ibr-7 can alter the expression of EGFR in pancreatic cancer cells is still unknown. In our study, Ibr-7 inhibited the expression of EGFR in pancreatic cancer cell lines, as we expected. EGFR inhibition could increase radiosensitivity of various tumours. Inhibition of EGFR/HER2 enhances radiosensitivity in pancreatic cancer [29, 30]. Targeting EGFR and the β1 integrin receptor efficiently induced radiosensitization in head and neck cancers. Based on these results, we further investigated the requirement of EGFR in Ibr-7-induced radiosensitivity in pancreatic cancer. Overexpression of EGFR decreased the apoptosis induced by Ibr-7 combined with radiation, which demonstrated the important role of EGFR in Ibr-7-mediated radiosensitization.

The AKT signalling pathway is associated with many tumour processes. Ibrutinib (10 μmol/L) could decrease p-AKT (S473) and its downstream genes, including mTOR and p-p70s6. However, Ibr-7 (2 μmol/L) did not significantly decrease the phosphorylation of AKT, mTOR and p70s6 (data not shown). As a result, the intrinsic mechanism remains to be further elucidated.

Radiation therapy leads to DNA lesions, and DSBs (DNA double strand breaks) are regarded as the major effect of radiation [31]. Radiosensitivity largely depends on the ability to repair radiation-induced DNA damage. γ-H2AX (the phosphorylated form of H2AX), which is induced by radiation, is involved in DNA damage and is closely associated with DSBs, serving as a sensitive and classic cellular indicator of DSBs [32, 33]. The number of γ-H2AX foci indicates the relative amount of DSBs. In our study, we found that Ibr-7 enhanced the number of γ-H2AX foci induced by radiation. In response to DNA damage, cell cycle progression is arrested. Our study found that Ibr-7 prolonged radiation-induced G2/M phase arrest, which was consistent with the higher level of γ-H2AX at 24 h in the Ibr-7 + radiation group than in the radiation-alone group. Based on these results, Ibr-7 may affect the expression of p-EGFR, leading to DNA damage in pancreatic cancer cells in response to radiation.

In conclusion, this study showed that Ibr-7 sensitized pancreatic cancer cells to radiation in vitro and that the effect was likely attributable, at least in part, to the induction of DNA damage, subsequent stimulation of G2/M phase arrest and cell apoptosis resulting from the downregulation of p-EGFR. To our knowledge, this is the first evidence showing Ibr-7-mediated radiosensitization in pancreatic cancer cells. Our preclinical results not only suggest an effective strategy to improve radiosensitivity of pancreatic cancer but also provide meaningful insights into the investigation of Ibr-7 in cancer treatment.

## Conclusions

In summary, Ibr-7 enhanced radiation sensitivity in pancreatic cancer cells by promoting cell cycle arrest at the G2/M phase, inducing cell apoptosis, and increasing DSBs induced by radiation. The mechanistic investigation revealed that a decrease in p-EGFR played an important role in the radiosensitizing effects of Ibr-7. This study indicates that Ibr-7 may be a novel radiosensitizer for pancreatic cancer.

## Supplementary information

**Additional file 1: Figure S1.** Ibr-7 and ibrutinib possessed potent anti-proliferative activity against pancreatic cancer cells. The dose- and time- dependent inhibitory effect of Ibr-7 (A, B) and ibrutinib (Ibr) (C, D) on two pancreatic cancer PANC-1 and Capan2 cell lines in vitro. Cells were treated with Ibr or Ibr-7 for 24, 48 or 72 h before CCK-8 assay.

**Additional file 2: Table S1.** The IC50 of Ibr-7 in PANC-1 and Capan2 cells.

**Additional file 3: Figure S2.** EGFR overexpression decreased cell apoptosis in pancreatic cancer cells in response to the combination treatment. (A) Both PANC-1 and Capan2 cells were transfected with EGFR plasmid and empty vector. Cells were collected after 24 h transfection and the expression of p-EGFR, EGFR were detected by western blot. (B) The ratio of EGFR/β-actin was quantified by densitometry based on immunoblot images. (C-D) The ratio of apoptosis in PANC-1 and Capan2 cells that had been transfected with EGFR plasmid or empty vector, then pretreated with Ibr-7 and exposed to radiation. The percentages of cell apoptosis were quantified. Results shown are the mean ± SD of 3 independent experiments. Significance was determined by Student’s t-test (*p < 0.05, **p < 0.01).

## Data Availability

All data generated or analyzed during this study are included in this published article and its additional files.
