# Supplementary figures and images for: The Ibr-7 derivative of ibrutinib radiosensitizes pancreatic cancer cells by downregulating p-EGFR
Source: Cancer Cell Int. 2020 Sep 17;20:458. doi: 10.1186/s12935-020-01548-6 (PMC7500014; doi:10.1186/s12935-020-01548-6)

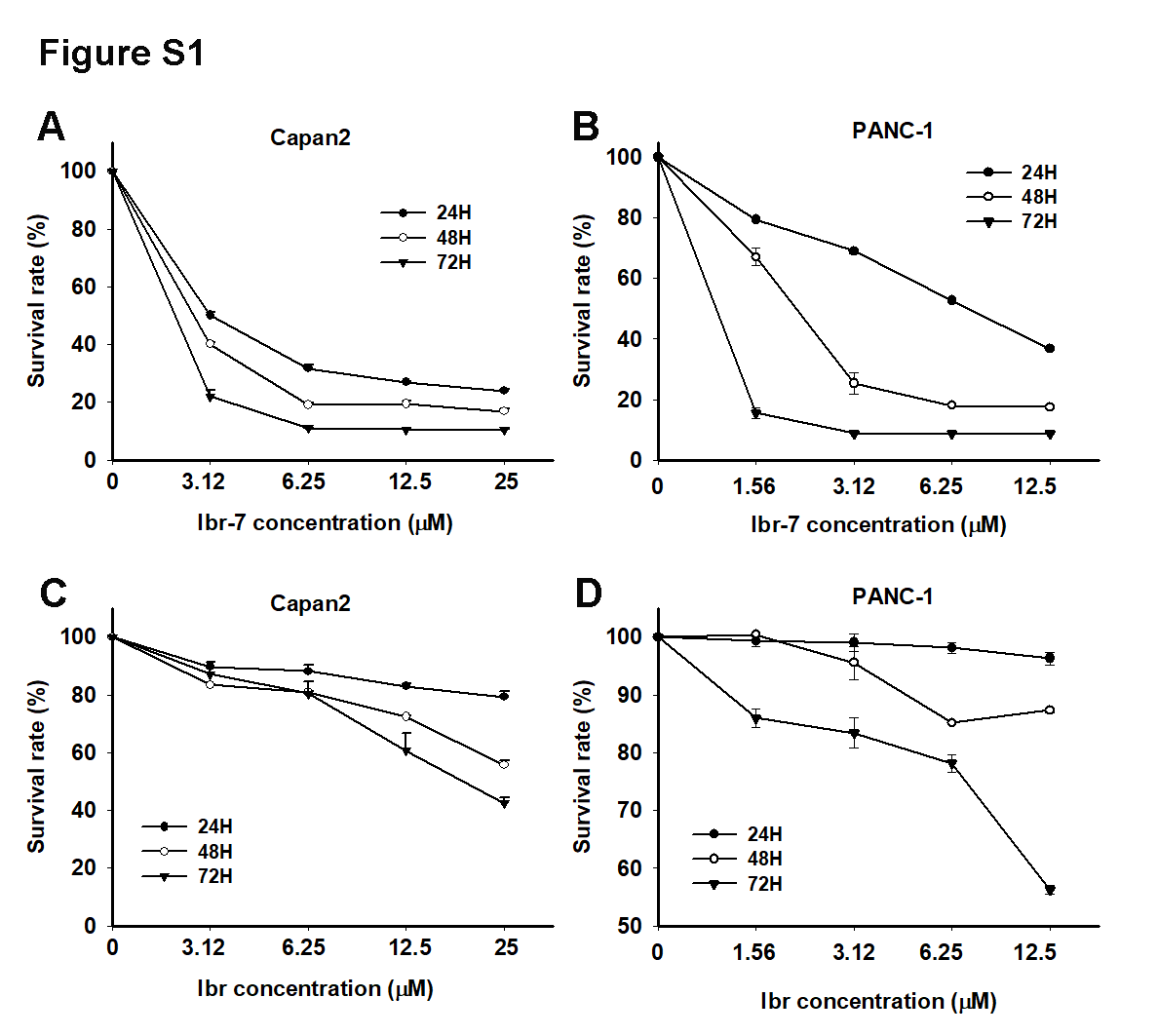

Supplement: Supplementary file 1 — Additional file 1: Figure S1. Ibr-7 and ibrutinib possessed potent anti-proliferative activity against pancreatic cancer cells. The dose- and time- dependent inhibitory effect of Ibr-7 (A, B) and ibrutinib (Ibr) (C, D) on two pancreatic cancer PANC-1 and Capan2 cell lines in vitro. Cells were treated with Ibr or Ibr-7 for 24, 48 or 72 h before CCK-8 assay. [file 12935_2020_1548_MOESM1_ESM.tif]

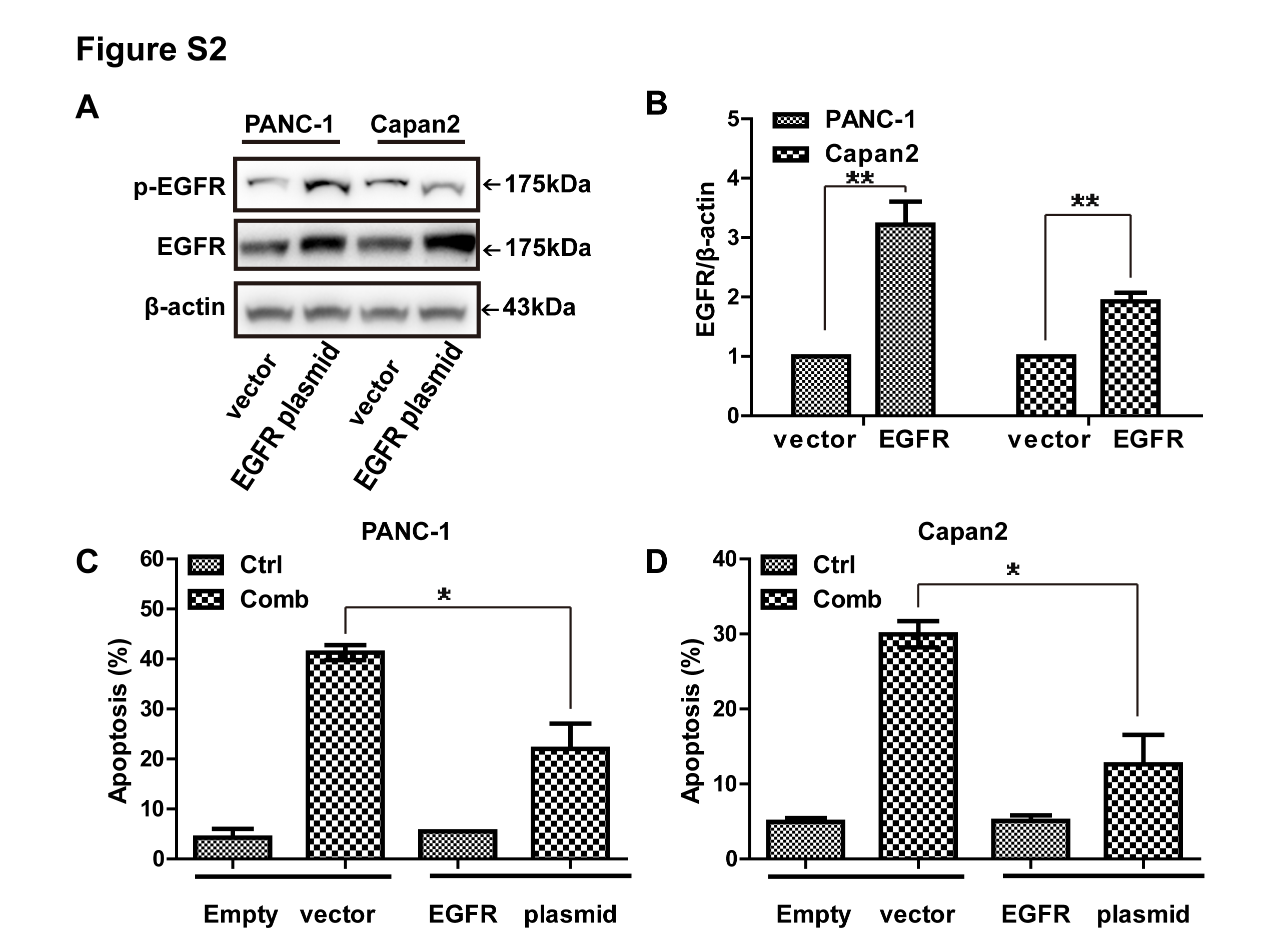

Supplement: Supplementary file 3 — Additional file 3: Figure S2. EGFR overexpression decreased cell apoptosis in pancreatic cancer cells in response to the combination treatment. (A) Both PANC-1 and Capan2 cells were transfected with EGFR plasmid and empty vector. Cells were collected after 24 h transfection and the expression of p-EGFR, EGFR were detected by western blot. (B) The ratio of EGFR/β-actin was quantified by densitometry based on immunoblot images. (C-D) The ratio of apoptosis in PANC-1 and Capan2 cells that had been transfected with EGFR plasmid or empty vector, then pretreated with Ibr-7 and exposed to radiation. The percentages of cell apoptosis were quantified. Results shown are the mean ± SD of 3 independent experiments. Significance was determined by Student’s t-test (*p < 0.05, **p < 0.01). [file 12935_2020_1548_MOESM3_ESM.tif]
