# Supplementary material for: The Ibr-7 derivative of ibrutinib radiosensitizes pancreatic cancer cells by downregulating p-EGFR
Source: Cancer Cell Int. 2020 Sep 17;20:458. doi: 10.1186/s12935-020-01548-6 (PMC7500014; doi:10.1186/s12935-020-01548-6)
Supplement: Supplementary file 2 — Additional file 2: Table S1. The IC50 of Ibr-7 in PANC-1 and Capan2 cells. [file 12935_2020_1548_MOESM2_ESM.docx]

Table S1 The IC50 of Ibr-7 in PANC-1 and Capan2 cells

| **Cell lines** | **24 H (μmol/L)** | **72 H (μmol/L)** |
| --- | --- | --- |
| PANC-1 | 5.1 | 1.2 |
| Capan2 | 2.6 | 2.0 |
